# MicroRNAs and cancer drug resistance: over two thousand characters in search of a role

**DOI:** 10.20517/cdr.2019.55

**Published:** 2019-09-19

**Authors:** Bruno Costa Gomes, José Rueff, António Sebastião Rodrigues

**Affiliations:** Centre for Toxicogenomics and Human Health; Genetics, Oncology and Human Toxicology, NOVA Medical School, Universidade Nova de Lisboa, Lisboa 1150-008, Portugal.

**Keywords:** MicroRNAs, gene regulation, cancer drug resistance, drug transporters, drug metabolism

## Abstract

MicroRNAs (miRNAs), a group of small regulatory noncoding RNAs, transformed our thinking on gene regulation. More than two thousand human miRNAs have been identified thus far. These bind imperfectly to the 3’-untranslated region of target mRNA and have been involved in several pathological conditions including cancer. In fact, major hallmarks of cancer, such as the cell cycle, cell proliferation, survival and invasion are modulated by miRNAs. Cancer drug resistance (CDR) has also been described as being modulated by miRNAs. CDR remains a burden for cancer therapy and patients’ outcome, often resulting in more aggressive tumours that tend to metastasize to distant organs. In this review we discuss the role of miRNAs influencing drug metabolism and drug influx/efflux, two important mechanisms of CDR.

## Introduction

Cancer drug resistance (CDR) is a burden in cancer therapy. It has social and economic consequences, and in most cases, it ends in patient death due to treatment failure. Roughly 90% of patients with metastatic cancers are confronted with treatment failure due to CDR. Drug resistance can be broadly classified into two types, intrinsic and acquired. Intrinsic drug resistance can be defined as a pre-existing genetic condition to a therapy approach that leads tumour cells to survive treatment, therefore contributing to therapy ineffectiveness from the beginning. This can be linked with drug breakdown, alteration of the drug target, resulting in a reduction of efficiency of targeted therapy, and altered drug transport. Acquired drug resistance is developed during therapy and usually due to adaptive processes, such as compensatory signalling pathways (reduced cell death and DNA damage response), drug inactivation, overexpression of drug targets, structural changes in drug targets, increased expression of drug efflux pumps and epigenetics^[1]^. Whatever the mechanisms of drug resistance, it results in treatment failure and consequent proliferation of resistant tumour cells that may metastasize and end up in patient death.

Combined actions of drug-metabolizing enzymes (DMEs) comprise Phase I and Phase II reactions of drug metabolism^[2,3]^. The former increases the polarity of drugs, followed by the conjugation reactions of Phase II that increase their polarity but block the reactivity of polar groups introduced in the earlier reaction. Subsequently, the resulting metabolite is effluxed through the membrane by ATP-binding cassette (ABC) transporters (Phase III reactions)^[4]^. Cancer cells tend to overexpress DMEs and transporters thus evading cancer treatment and becoming resistant to several drugs.

The term microRNAs (miRNAs) was first defined in 2001 by Lee and Ambros^[5]^. However, they were first described in 1993 when two independent groups^[6,7]^ published experiments on the Caenorhabditis elegans *lin-4* gene which codes for a pair of small RNAs with antisense complementarity to multiple sites on the 3’-untranslated region (UTR) of the *lin-14* gene. Subsequently, they were shown to act on several key cellular processes, such as cell differentiation, cell cycle progression, and apoptosis. Thus, miRNAs can be defined as short (approximately 22 nucleotides) non-coding RNAs that regulate gene expression by binding to the 3’-UTR of messenger RNA. The small size of miRNAs and the pairing between a miRNA and a target site that does not need to be perfect results in a wide selection of genes that can be subject to regulation. Indeed, one miRNA can regulate the expression of multiple mRNAs with wide effects in the transcriptome^[8]^, besides possible regulatory effects on other miRNAs, forming a circuitry of epigenomic regulation. However, the property that makes miRNAs versatile also hampers the prediction of putative targets and the conclusive mechanisms of regulation in the cell. Thus, the study of miRNAs can be very complex. Due to their characteristics and their broad influence in cell homeostasis, soon after their discovery, miRNAs were associated with cancer^[9]^ and referred to as possible regulators of drug resistance^[10]^. More than two thousand human miRNAs have been identified thus far. To date, several studies have shown that drug resistance is influenced by miRNAs. This review intends to summarize the miRNAs that have been shown to regulate drug uptake proteins and Phase I, II and III drug metabolism in different tumours and the corresponding drugs for which the tumours are resistant.

## Drug uptake proteins and miRNAs

**Figure 1:**
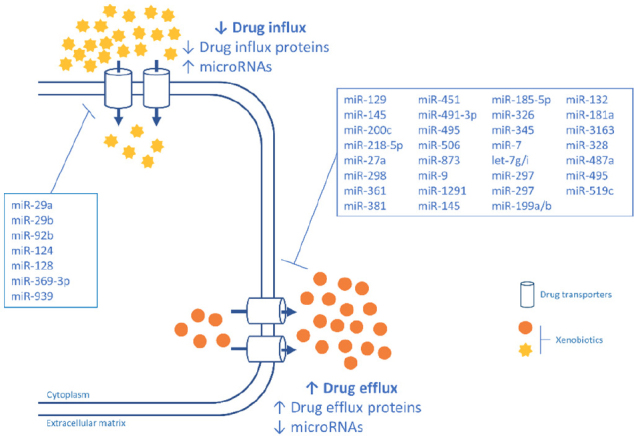
A schematic representation of the regulation of influx and efflux proteins by miRNAS

Several cancer drugs are absorbed by intestinal epithelial cells which express a variety of influx transporters that are specific for drugs, amino acids, peptides, organic anions and cations, and other nutrients. These transporters are differentially expressed in different regions in the intestine. Peptide transporter 1 (PEPT1/ SLC15A1), organic cation/carnitine transporter 2 (SLC22A5), organic anion transporting polypeptide 2B1 (SLCO2B1), and monocarboxylate transporter 1 (MCT1/SLC16A1) are expressed at the brush-border membrane, whereas organic cation transporter 1 (SLC22A1) is mainly expressed at the basolateral membrane in the small intestine^[11]^. Recent studies have indicated that miRNAs contribute to the differentiation and viability of the intestinal epithelium, and the regional differences in the expression of these transporters in the intestine are dependent on the differentiation of intestinal epithelial cells^[12]^. Thus, abnormal expression of miRNAs can have a clear impact on absorption of several drugs. We summarize in Table 1 the miRNAs that regulate uptake proteins in cell membranes. Figure 1 shows a schematic representation of the regulation of influx and efflux proteins by miRNAS.

**Table 1:** Drug uptake proteins and miRNAs that regulate their expression

Target	miRNA	Model	Drug	Reference
SLC15A1	miR-92b	Caco2-BBE cells	NS	Dalmasso *et al*.^[13]^, 2011
SLC16A1	miR-29b	mhAT3F, MIN6, and HEK293 cells		Pullen *et al*.^[14]^, 2011
	miR-29a			Pullen *et al*.^[14]^, 2011
	miR-124			Pullen *et al*.^[14]^, 2011
SLC34A2	miR-939	gastric cancer cell lines and tissue	5-fluorouacil	Zhang *et al*.^[15]^, 2017
SLC35F5	miR-369-3p	NSCLC cells, 16HBE, and HEK293T	cisplatin	Hao *et al*.^[16]^, 2017
GLUT1	miR-128	bladder cancer cells (T24 and EJ)	cisplatin	Li *et al*.^[17]^, 2017

NS: not stated

## SLC15A1, SLC16A1 and SLC34A2

SLC15A1 was shown to be regulated by miR-92b, causing a decrease in the expression of the uptake transporter^[13]^. Pullen *et al*.^[14]^ showed that miR-29a, miR-29b, and miR-124 regulate SLC16A1, without being its main regulator. The authors refer that these miRs might be a complement mechanism in SLC16A1 expression. Unfortunately, both authors failed to associate these mechanisms with specific drugs and consequent drug resistance. SLC34A2 was shown to be regulated by miR-939 in gastric cancer cell lines and tissues and miR-939 was associated with CDR in gastric cancer patients^[15]^. miR-939 inhibited gastric cancer metastasis and enhanced the sensitivity of gastric cancer cells to 5-fluorouracil treatment, although uptake of 5-fluorouracil is known to be predominantly by diffusion. Nevertheless, by using multivariate analysis, the authors could show that the combination of both miR-939 and SLC34A2 are indicators of poor prognosis and tumour recurrence in gastric cancer patients, pointing that miR-939 inhibits the SLC34A2/Raf/MEK/ERK pathway, which is activated in gastric cancer.

## SLC35F5

Hao *et al*.^[16]^ showed that miR-369-3p may regulate cisplatin chemoresistance by directly targeting the 3’-UTR of SLC35F5. miR-369-3p acts as an oncogenic miRNA since it is highly expressed in non-small cell lung cancer cells and consequently negatively regulates SLC35F5. Thus, cisplatin is not internalized by the cell and consequently its therapeutic action fails.

## GLUT1

Regarding GLUT1 our search only retrieved one study where miR-128 regulates GLUT1^[17]^. The authors showed that the overexpression of miR-128 lowered the rate of glucose uptake and the total level of GSH. It also enhanced the sensitivity of bladder cancer to cisplatin.

## Phase I proteins and miRNAs

Various studies have been performed on transcriptional and translational regulation of the DMEs^[18-20]^. However, these studies have not elucidated the mechanisms of their post-transcriptional regulation. MiRNAs are regulators of DMEs, however, few studies have shown a direct involvement of this regulation in CDR^[21]^.

One of the key players of drug metabolism are cytochrome P450 (CYP) enzymes that catalyse oxidation reactions of xenobiotics^[22]^. More than 90% of the reactions involved in the metabolism of all endobiotics and xenobiotics are catalysed by P450s^[23]^. The majority of CYP reactions are catalysed by a set of four CYP families: 1A, 2C, 2D, and 3A, with the largest fraction of the CYP reactions being catalysed by CYP3A enzymes. One of the most important CYPs is CYP3A4, which metabolizes from 13% of general chemicals to 27% of drugs^[23]^. Consequently, regulation of DMEs is crucial to drug efficacy and is associated with drug failure and/or drug resistance. In Table 2 we show the known miRNAs that regulate CYPs and influence CDR.

**Table 2:** Phase I DMEs and miRNAs that regulate their expression

Target	miRNA	Model	Drug	Reference
CYP1A2	miR-132-5p	HepaRG cells	Lansoprazole	Chen *et al*.^[26]^, 2017
		Huh-7 cells		
		HepG2 cells		
		HepG2 cells	Flutamide	
CYP1B1	miR-187-5p	NSCLC	NS	Mao *et al*.^[35]^, 2016
		A549		
		SPC-A-1		
	miR-27b	HeLa cells	NS	Tsuchiya *et al*.^[31]^, 2006
		MCF-7 cells		
		Jurkat cells		
		HepG2 cells	Fumonisin B_1_	Chuturgoon *et al*.^[33]^, 2014
	miR-200c	Caki-1 cells	Docetaxel	Chang *et al*.^[34]^, 2015
		Caki-2 cells		
		A498 cells		
		ACHN cells		
		786-O cells		
		769-P cells		
CYP2E1	miR-552	PLC/PRF/5 cells	NS	Miao *et al*.^[40]^, 2016
		HepG2 cells		
		C57/BL6 mice		
	miR-378	HEK293		Mohri *et al*.^[38]^, 2010
	miR-132	Primary Rat Hepatocytes	Rapamycin	Shukla *et al*.^[39]^, 2013
	miR-212			
CYP3A4	miR-27b	Human liver tissue	Atorvastatin	Liu *et al*.^[43]^, 2016
	miR-206			
	miR-27b	[C1 cells LS-180 cells	Cyclophosphamide	Pan *et al*.^[42]^, 2009

NS: not stated; DMEs: drug metabolizing enzymes

## CYP1A2

CYP1A2 is an important DME since it represents 13% of all CYPs expressed in the liver and metabolizes about 5% of currently used drugs. CYP1A2 is also important in the metabolism of endobiotics like steroids and environmental pollutants like polycyclic aromatic hydrocarbons. Several genetic polymorphisms have been associated with increased activity and linked with lung cancer^[24,25]^. Regarding post-translational regulation of CYP1A2, one study reported that miR-132-5p decreases *CYP1A2* gene expression and influences hepatic cells in the metabolisation of lansoprazole and flutamide^[26]^. The authors showed direct targeting of miR-132-5p to CYP1A2 and also demonstrated that the decreased expression of CYP1A2 attenuates lansoprazole- and flutamide mediated toxicity.

## CYP1B1

CYP1B1 is highly expressed in oestrogen target tissues and catalyses the metabolic activation of several procarcinogens (e.g., heterocyclic amines, polycyclic hydrocarbons) and the 4-hydroxylation of 17β-oestradiol^[27,28]^. It is also abundant in tumour tissues. It was already shown that polymorphisms in CYP1B1 can influence its activity and thus are associated with cancer, namely breast cancer^[29]^. *CYP1B1* gene expression can also be modulated by aryl hydrocarbon receptor, an important mediator of toxic response and consequently drug efficacy^[30]^. In what concerns post-transcriptional regulation, a few authors have shown that miRNAs can influence *CYP1B1* gene expression. One of the first authors showing this association was the group of Tsuchiya *et al*.^[31]^ who validated miR-27b as a regulator of CYP1B1 in cervical and breast cancer cell lines, and also reported an inverse association of miR-27b expression and CYP1B1 protein expression in breast tissue samples. These authors showed through immunohistochemistry that miR-27b decreased gene expression together with strong CYP1B1 tissue staining. However, the authors did not show an association of this pattern with common breast CDR. Nevertheless, since biotransformation of tamoxifen, a widely used drug in breast cancer treatment, occurs via CYP1B1^[32]^, the mis-expression of CYP1B1 in breast cancer cells due to miR-27b could influence the efficacy of tamoxifen. Interestingly, Chuturgoon *et al*.^[33]^ also reported the involvement of miR-27b in the metabolisation of fumonisin B1, a known mycotoxin, through direct targeting of CYP1B1 and hepatic neoplastic transformation, reinforcing the idea that miRNAs and CYPs interact in the metabolisation of drugs and environmental and food contaminants. Recently, miR-200c was also predicted to be involved in CYP1B1 regulation in renal cell cancer and resistance to docetaxel^[34]^. The authors used several renal cancer cell lines to show this association and proved that miR-200c directly targets CYP1B1 and the low expression of miR-200c in these cell lines is correlated with an increased expression of CYP1B1. More recently, Mao *et al*.^[35]^ showed that miR-187-5p is decreased in non-small lung cancer and regulates CYP1B1, a direct target of miR-187-5p. The authors used lung cancer cell lines to demonstrate the negative correlation of both miR-187-5p and CYP1B1 and show its direct targeting. They also showed a correlation between the low expression of miR-187-5p with TNM stage and postoperative survival, and high expression of miR-187-5p with growth and metastasis. However, the authors failed to associate these results with drug resistance.

## CYP2E1

CYP2E1 represents approximately 7% of total CYPs in the human liver. CYP2E1 catalyses the metabolism of several low molecular weight xenobiotics, such as organic solvents (e.g., ethanol, acetone, and chloroform), and several procarcinogens (e.g., N-nitrosodimethylamine and N-nitrosomethylethylamine). Interestingly, CYP2E1-induced ROS generation influences migration in breast cancer cells, thus may be involved in breast cancer metastasis^[36]^. Genetic polymorphisms have been associated with CYP2E1 efficiency and influence lipid metabolism and nicotine clearance in the blood^[37]^. Regarding epigenetic regulation, miR-378 was confirmed as a CYP2E1 regulator in kidney cell lines by Mohri *et al*.^[38]^. The authors showed that the overexpression of miR-378 significantly decreased CYP2E1 protein levels and enzyme activity. An interesting detail is that miR-378 did not enable the degradation of the CYP2E1 mRNA. Additionally, an inverse association of the expression levels of miR-378, CYP2E1 mRNA and protein as well as enzyme activity were revealed using a panel of 25 human livers. Unfortunately, the authors did not show any association with cancer drug failure. However, other authors showed an association of an increased expression of CYP2E1, low expression of miR-132 and miR-212 with rapamycin resistance in cultured rat hepatocytes^[39]^. More recently, miR-552 was shown to regulate CYP2E1^[40]^. This study had the meticulousness of showing that miR-552 can influence CYP2E1 expression in a transcriptional and post-transcriptional manner. In fact, the authors showed that a non-seed region regulation by miR-552 can occur, thus influencing transcription and translation independently. However, the authors did not show any association with cancer drugs.

## CYP3A4

CYP3A4 represents 30% of the CYP expression in the liver and metabolizes approximately 27% of all commercial drugs. For that reason, it is one of the most studied CYPs. As with the other CYPs, it has several isoforms that results from genetic polymorphisms^[41]^. Some authors showed that CYP3A4 can be regulated post-transcriptionally by miR-27b^[42]^. These authors also showed that an overexpression of miR-27b in the PANC1 cell line led to a lower sensitivity to cyclophosphamide, showing the impact on drug response and resistance. Other authors also showed that miR-27b can influence the metabolism of atorvastatin in the liver, a known statin used to low cholesterol levels in the blood^[43]^. Moreover, these authors also showed in the same study that miR-206 can regulate CYP3A4 and influence atorvastatin metabolism. This study was important to understand the mechanism of atorvastatin resistance, since it can affect 60% of the patients. Although statin is not a common cancer drug, a recent study performed in ovarian cancer^[44]^ showed that atorvastatin has a role in proliferation and metastasis. In fact, the authors showed that atorvastatin inhibited cell proliferation of ovarian cancer cells in a dose-dependent manner and that its anti-proliferative activity was linked with induction of apoptosis, autophagy, cellular stress and cell cycle arrest via AKT/mTOR and MAPK pathways. Also, atorvastatin changed cell adhesion and invasion and decreased expression of VEGF and MMP9, known important proteins in epithelial-to-mesenchymal transition^[44]^.

## Phase II enzymes and miRNAs

**Figure 2:**
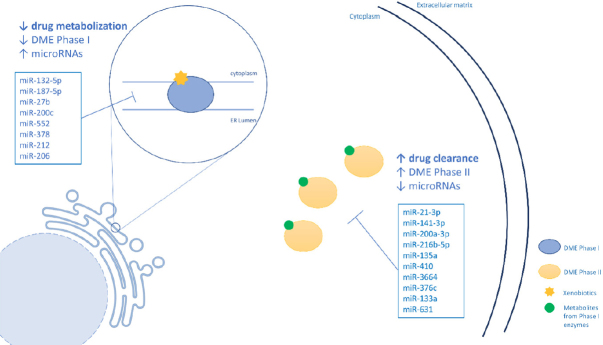
A schematic representation of the regulation of DMEs by by miRNAS. DMEs: drug metabolizing enzymes

Regarding Phase II DMEs, although these enzymes are important players in cancer drug detoxification, few studies have linked them with miRNAs regulation and CDR. A summary of the miRNAs that regulate Phase II enzymes can be seen in Table 3. Figure 2 shows a schematic representation of the regulation of DMEs by miRNAS.

**Table 3:** DMEs of Phase II and miRNAs that regulate their expression

Target	miRNA	Model	Drug	Reference
UGT1A1	miR-21-3p	NS	NS	Papageorgiou *et al*.^[53]^, 2017
	miR-141-3p			
	miR-200a-3p			
UGT2B4	miR-216b-5p	HepG2	Epirubicin	Dluzen *et al*.^[51]^, 2016
	miR-135a	NS	NS	Wijayakumara *et al*.^[54]^, 2017
	miR-410			
UGT2B7	miR-3664			
UGT2B10	miR216b-5p	Liver cancer	Epirubicin	Dluzen *et al*.^[51]^, 2016
UGT2B17	miR-376c	NS	NS	Margaillan *et al*.^[52]^, 2016
GSTP1	miR-133a	Head and neck, oesophageal, bladder	Cisplatin and carboplatin	Moriya *et al*.^[50]^, 2012
SULT1A1	miR-631	Breast cancer	Actinomycin D	Yu *et al*.^[47]^, 2010

NS: not stated; DMEs: drug metabolizing enzymes

## SULT1A1

SULT1A1 is a member of the sulfotransferase (SULT) family, which catalyse the transfer of the sulfonyl group from 3’-phosphoadenosine 5’-phosphosulfate (PAPS) to nucleophilic groups of a variety of xenobiotic and endogenous compounds, thus increasing their solubility and excretion^[45]^. SULT1A1 is the most highly expressed SULT in the liver, and several therapeutic agents, such as 4-hydroxytamoxifen, are substrates of SULT1A1. Variability in the activity levels of SULT1A1 can influence the efficacy of some drugs and consequently induce drug resistance^[46]^. Interestingly, genetic polymorphisms in SULT1A1, such as copy number variation and single nucleotide polymorphisms (SNPs), are associated with altered enzymatic activity. In fact, some authors^[47]^ studied SNPs in the 3’-UTR region of SULT1A1 and in silico analyses predicted that the 973C→T SNP could influence the binding of miR-631. Taking this into account, *in vitro* luciferase reporter assays and overexpression of miRNAs inhibitors in ZR75-1, MCF7, and MCF10A breast cell lines confirmed that SULT1A1 is a direct target of miR-631^[47]^.

## GSTP1

GSTP1 is a member of the GST enzyme superfamily, that catalyses the conjugation of electrophiles with glutathione in Phase II reactions, including platinum drugs such as cisplatin and carboplatin^[48]^. GSTP1 plays several roles in cells, such as in stress responses, signalling, and apoptosis. GSTP1 knockdown selectively influenced cisplatin and carboplatin chemosensitivity, cell invasion and migration^[49]^. Overexpression of GSTP1 has been observed in many types of cancer and cell lines either inherently or made resistant to chemotherapy drugs, including cisplatin and various alkylating agents. As other DMEs, GSTP1 can be regulated by miRNAs and Moriya *et al*.^[50]^ showed that reduced expression of miR-133a leads to an increased expression of GSTP1, contributing to drug resistance. The authors showed that the transfection of miR-133a repressed GSTP1 expression at both mRNA and protein levels in several different cell lines. Thus, the functional significance of miR-133a was investigated using head and neck squamous cell carcinoma (SCC), oesophageal SCC, and bladder cell lines. These authors could show that restoration of miR-133a expression inhibited cancer cell proliferation, invasion, and migration, suggesting that miR-133a may function as a tumour suppressor.

More recently, some studies were published showing the importance of miRNAs in the regulation of UDP-glucuronosyltransferases (UGTs) enzymes^[51-54]^. The UGTs are critical for the efficient elimination of several drugs, including cancer drugs.

## UGT1A

The UGT1A subfamily is involved in the metabolism of more than half of the drugs eliminated by glucuronidation. Among other examples, we can refer irinotecan, raltegravir and muycophenolic acid, drugs involved in cancer therapy, HIV and organ rejection, respectively. Recently, some authors showed that UGT1A1 can be regulated by miR-21-3p, miR-141-3p and miR-200a-3p^[53]^. Although the authors did not show any association with drug resistance, they demonstrated that polymorphisms on the 3’-UTR region of UGT1A1 can influence the targeting of these miRNAs and consequently its activity, namely, rs10929303, rs1042640, and rs8330 polymorphisms.

## UGT2B

The UGT2B subfamily is responsible for metabolic clearance of several endobiotics, such as bile acids and steroid hormones, and xenobiotics, such as cancer drugs^[55]^. In fact, Dluzen *et al*.^[51]^ showed that miR-216b-5p can influence epirubicin treatment efficacy by targeting UGT2B4 and UGTB10 in liver cancer. Interestingly, the approach of these authors was similar to the previous one that studied UGT1A1, studying the 3’-UTR region of UGT2B isoforms and associating these variants with miRNAs hybridization to messenger RNA. Other authors also showed that UGT2B4 can be regulated by miR-216b-5p and miR-135a^[54]^, although not necessarily associated with CDR. Another isoform of UGT2B, UGT2B7, was shown to be regulated by miR-3664^[54]^. Finally, miR-376c was showed to regulate UGT2B17^[52]^. Once more, the authors failed to show an association with drug resistance but showed a possible association with steroid metabolism and prostate cancer.

## Phase III proteins and miRNAs

Phase III proteins are known to be responsible for drug transport through cell membranes. It is a critical step in allowing access of some compounds to intracellular targets. For this reason, the involvement of drug transport is one of the most studied mechanisms in CDR^[56]^. Multidrug resistance (MDR) is frequently linked to overexpression of one or more of drug transporters. One of these phase III proteins are the ABC transporters, which have an important cellular role in the efflux of several endobiotics and xenobiotics^[57]^. Approximately 50 different ABC transporters have been identified and classified in seven families from ABCA through ABCG^[58]^. The relevance of miRNAs in the regulation of ABC transporters has been reviewed before^[11,59,60]^, and probably are the most studied drug metabolism proteins in what concerns miRNAs regulation. In Table 4 we summarize all miRNAs described as being involved in drug resistance by regulation ABC transporters.

**Table 4:** Drug transporters and miRNAs that regulate their expression

Target	miRNA	Model	Drug	Reference
ABCB1	miR-129	ovarian	paclitaxel	Wang *et al*.^[68]^, 2018
		gastric cancer	cisplatin	Lu *et al*.^[69]^, 2017
	miR-145	colon carcinoma, kidney	NS	Ikemura *et al*.^[73]^, 2013
	miR-200c	Breast Cancer	doxorubicin	Chen *et al*.^[76]^, 2012
	miR-218-5p	gallbladder	gemcitabine	Wang *et al*.^[74]^, 2017
	miR-27a	Ovarian cancer	doxorubicin	Zhu *et al*.^[79]^, 2008
	miR-298	Breast Cancer	doxorubicin	Bao *et al*.^[77]^, 2012
	miR-361	gastric cancer	oxaliplatin	Wu *et al*.^[70]^, 2018
	miR-381	Breast Cancer	cisplatin	Yi *et al*.^[80]^, 2019
		K562 cell line	adriamycin	Xu *et al*.^[81]^, 2013
	miR-451	Breast Cancer	doxorubicin	Kovalchuk *et al*.^[78]^, 2008
	miR-491-3p	hepatocellular carcinoma	doxorubicin & vinblastin	Zhao *et al*.^[71]^, 2017
	miR-495	ovarian cancer & gastric cancer	doxorubicin & taxol	Zou *et al*.^[83]^, 2017
		K562 cell line	adriamycin	Xu *et al*.^[81]^, 2013
	miR-506	colorectal cancer	oxaliplatin	Zhou *et al*.^[72]^, 2017
	miR-873	ovarian cancer	cisplatin & paclitaxel	Wu *et al*.^[75]^, 2016
	miR-9	chronic myelogenous leukemia	adriamycin	Li *et al*.^[82]^, 2017
ABCC1	miR-1291	pancreatic cancer, lung cancer, kidney	doxorubicin	Pan *et al*.^[88]^, 2013
	miR-145	gallbladder	cisplatin	Zhan *et al*.^[89]^, 2016
		Breast Cancer	doxorubicin	Gao *et al*.^[90]^, 2016
	miR-185-5p	non-small cell lung cancer	cisplatin	Pei *et al*.^[91]^, 2016
	miR-326	MCF-7 cells	VP-16 and doxorubicin	Liang *et al*.^[87]^, 2010
	miR-345	MCF-7 cells	cisplatin	Pogribny *et al*.^[86]^, 2010
	miR-7	MCF-7 cells	cisplatin	Pogribny *et al*.^[86]^, 2010
ABCC10	let-7g/i	esophageal carcinoma	cisplatin	Wu *et al*.^[75]^, 2016
ABCC2	miR-297	HCT116 cells	oxaplatin and vincristine	Xu *et al*.^[92]^, 2012
ABCG2	mi-199a/b	colorectal cancer	cisplatin	Chen *et al*.^[102]^, 2017
	miR-132	gastric cancer	cisplatin	Zhang *et al*.^[103]^, 2017
	miR-181a	Breast Cancer	MX	Jiao *et al*.^[98]^, 2013
	miR-3163	Retinoblastoma Cancer	cisplatin, carboplatin, vincristine, doxorubicin, and etoposide	Jia *et al*.^[104]^, 2016
	miR-328	Breast Cancer	MX	Li *et al*.^[99]^, 2011
		kidney, breast cancer	MX	Pan *et al*.^[100]^, 2009
	miR-487a	MCF-7 cells	MX	Ma *et al*.^[101]^, 2013
	miR-495	non-small cell lung cancer	cisplatin	Guo *et al*.^[105]^, 2018
	miR-519c	kidney, breast cancer	MX	Li *et al*.^[99]^, 2011

NS: not stated; MX: mitoxantrone

## ABCB1

ABCB1 is probably the most studied ABC transporter. It is also known as MDR1 or P-gp transporter and is often overexpressed in tumour cells with chemotherapeutic resistance, as well as in resistant cancer cell lines^[61]^. An overexpression or altered function due to genetic polymorphisms of ABCB1 increases resistance to taxanes (e.g., paclitaxel and docetaxel)^[62]^, epipodophyllotoxin derivatives (e.g., etoposide and teniposide)^[63]^, anthracyclines (e.g., doxorubicin)^[63,64]^, antibiotics (e.g., actinomycin D)^[63,65]^, vinca alkaloids (e.g., vinblastine and vinorelbine)^[66]^, and tyrosine kinase inhibitors (e.g., imatinib and dasatinib)^[67]^.

To date, several studies have been published reporting a correlation between miRNAs expression and ABCB1. Wang *et al*.^[68]^ showed that ABCB1 was a direct target of miR-129 and that urothelial carcinoma associated 1 (UCA1) de-repressed ABCB1 expression by sponging miR-129. Thus, the authors revealed an interesting regulatory axis UCA1/miR-129/ABCB1 that sensitizes ovarian cancer cells to paclitaxel. In another study, it was shown that miR-129 also sensitizes cancer cells to cisplatin, namely in gastric cancer, by regulating ABCB1^[69]^. The confirmation of direct targeting was done in BGC823/DDP and MKN45/DDP cell lines resistant to cisplatin that presented low expression of miR-129. The authors also confirmed that miR-129 expression was significantly downregulated and ABCB1 upregulated in gastric cancer tissues of cisplatin-resistant patients. Other authors also reported this sponging activity by another long non-coding RNA, BLACAT1, which regulates miR-361 expression and consequently increases ABCB1 expression. This regulatory axis BLACAT1/miR-361/ABCB1 can influence resistance to oxaliplatin^[70]^. Albeit indirectly, miR-491-3p was shown to regulate Sp3, a transcription factor of ABCB1, in hepatocellular carcinoma and consequently doxorubicin and vinblastine resistance. The authors observed this regulatory axis miR-491-3p/Sp3/ABCB1 in cell lines and tissue from cancer patients^[71]^. Similarly, miR-506 seems to enter a regulatory axis with the Wnt/β-catenin pathway which regulates ABCB1. This was demonstrated in colorectal cancer cell lines and helped clarify the resistance mechanism of oxaliplatin^[72]^.

Ikemura *et al*.^[73]^ showed that miR-145 regulates ABCB1 expression in intestinal epithelial cells and kidney cells. The authors did not show any association with chemotherapeutics but instead showed an increased efflux activity with rhodamine 123. In another study^[74]^ miR-218-5p was shown to indirectly regulate ABCB1 by inhibiting the translation of PRKCE, a member of the protein kinase C family, which is known as an ABCB1 activator. The authors used an *in vitro* and *in vivo* approach to show these results and in fact, were capable of inducing gemcitabine sensitization in gallbladder cancer in both models. With the same approach, other authors^[75]^ could show that miR-873 can mediate resistance to paclitaxel and cisplatin in ovarian cancer.

In one study with breast cancer, a correlation of miR-200c with poor response to neoadjuvant chemotherapeutics could be shown^[76]^. Unfortunately, the authors did not follow ABCB1 mRNA and protein expression in the patients. Instead, they used a doxorubicin-resistant breast cancer cell line (MCF7/ADR) to prove that miR-200c regulates ABCB1 by directly targeting its 3’-UTR. Curiously, miR-200c expression was observed to be downregulated over 800-fold in this resistant cell line. In two other studies^[77,78]^ the importance of miRNAs in the resistance of doxorubicin in breast cancer was shown, namely, miR-298 and miR-451. Kovalchuk *et al*.^[78]^ showed that the ABCB1 is highly expressed in the MCF-7-resistant breast tumour cell line when compared with wild type MCF-7, and that a negative correlation exists between ABCB1 and miR-451 expression. Transfection of miR-451 re-established the sensitivity of the MCF-7-resistant cells to doxorubicin. Conversely, a decreased expression of miR-451 is correlated with higher expression of ABCB1 in other drug resistant cells, more precisely in a human ovarian cancer cell line and a human cervix carcinoma cell line. Here, the expression of miR-27a and miR-451 were upregulated in multidrug resistant cells compared with their parental lines, downregulating expression of the *ABCB1* gene^[79]^. These results suggest that the involvement of specific miRNAs in drug resistance should be taken cautiously, since the results could depend on various factors, including the cell lines under study. Bao *et al*.^[77]^ used a different breast tumour cell line, MDA-MB-231, to show that miR-298 regulates *ABCB1* gene expression and increases resistance to doxorubicin. Remarkably, the authors also showed that miRNAs processing is altered in the resistant cell lines, since DICER is weakly expressed and higher levels of miR-298 precursor was detected instead of the mature form. Also in breast cancer, miR-381 overcomes cisplatin resistance by targeting ABCB1^[80]^. Their results showed that miR-381 was down-regulated in cisplatin resistant breast cancer tissues and cell lines, and ABCB1 expression is inversely correlated. The authors observed an interaction between miR-381/ABCB1 and showed that inhibition of miR-381 reduced sensitivity of MCF-7 and MDA-MB-231 cells to cisplatin. MDR1 knockdown could overcome cisplatin resistance in MCF-7 and MDA-MB-231 cisplatin resistant cells, while MDR1 overexpression led to DDP resistance in MCF-7 and MDA-MB-231 cells. Thus, a biological interaction between both was demonstrated. miR-381 seems also to regulate adriamycin resistance in chronic myelogenous leukaemia K562 cell lines^[81]^. Functional analysis indicated that restoring expression of miR-381 and also miR-495 in K562 adriamycin-resistant cells was associated with an inverse expression of ABCB1 and consequently increased drug accumulation in the cells and drug sensitisation. Other authors^[82]^ also showed that miR-9 can have the same effect in adriamycin resistance. Another study^[83]^ showed that miR-495 can sensitize ovarian and gastric cancer cell lines to a mixture of taxol and doxorubicin. The authors started showing that resistant cell lines can tolerate a high dosage of this drug mixture and have increased levels of ABCB1. Later, they showed that miR-495 is inversely correlated with ABCB1 and miR-495 expression induction lowered ABCB1 expression levels and sensitized cells to taxol and doxorubicin. This pattern was also shown *in vivo*.

## ABCC1, ABCC2 and ABCC10

The ABCC transporter family needs the presence of glutathione for its activity and ABCC1 and ABCC2 (also known as MRP1 and MRP2) share 49% amino acid residues. Altered ABCC1 activity is known to confer resistance to vincristine, etoposide, anthracyclines (doxorubicin, daunorubicin, epirubicin), mitoxantrone (MX), flutamide, and methotrexate^[84]^. ABCC2 can mediate resistance to methotrexate, cisplatin, irinotecan, paclitaxel, and vincristine. Both ABCC1 and ABCC2 are mainly expressed in solid tumours from the kidney, colon, breast, lung, ovary, and as well as in cells from patients with acute myelogenous leukaemia^[85]^. ABCC10 (also known as MRP7) is genetically more distant (33%-36%) and is also expressed in several tissues. It can modulate resistance to paclitaxel, docetaxel, vincristine, vinblastine, Ara-C, gemcitabine and epothilone-B^[85]^.

Regarding ABCC1, various studies were published showing a regulation by miRNAs. Pogribny *et al*.^[86]^ showed that miR-345 and miR-7 increase sensitivity to cisplatin in breast cancer cells. The authors used a MCF-7 cell line resistant to cisplatin which expresses high levels of ABCC1 and lower levels of miR-345 and miR-7. Also in breast cancer, other authors showed that miR-326 represses ABCC1 expression and sensitizes VP-16 resistant MCF-7 cells to VP-16 and doxorubicin^[87]^. Another study reported that miR-1291 is originated from a nucleolar RNA, SNORA34, and influences doxorubicin resistance in pancreatic cancer, lung cancer, kidney cancer^[88]^. More recently, miR-145 was shown to regulate cisplatin resistance in gallbladder cancer cell lines by targeting ABCC1. Lower miR-145 and higher ABCC1 expression levels in gallbladder tissue predicted poor prognosis of gallbladder cancer patients who received chemotherapy^[89]^. Another microRNA, miR-145, was also reported to modulate doxorubicin resistance by targeting ABCC1 in an *in vitro* and *in vivo* study^[90]^. miR-185-5p was shown to control cisplatin resistance via ABCC1 in non-small cell lung cancer. This inverse association was only demonstrated in lung cancer cell lines A549 resistant to cisplatin, thus, lacking data in lung cancer tissue^[91]^.

Regarding ABCC2, only one study has been published showing an inverse correlation of miRNA with ABCC2. Xu *et al*.^[92]^ reported the validation of ABCC2 as a target of miR-297 and consequently the downregulation of its expression. An inverse correlation was demonstrated in colorectal carcinoma cell lines resistant to oxaliplatin and vincristine. After transfection of miR-297-mimics, *in vitro* and *in vivo*, these cancer cells overcame resistance to oxaliplatin.

Regarding ABCC10 also one article has reported its modulation by miRNAs. In this study, miR-let-7g/i was shown to hinder ABCC10 translation in oesophageal carcinoma and thus influencing cisplatin resistance. The authors also refer a regulation of proliferation and apoptosis possibly through interaction with BAG3, a protein involved in several cellular processes. However, this assumption needs confirmation with further studies^[93]^.

## ABCG2

ABCG2 is mainly expressed in the gut, bile canaliculi, placenta, blood-testis and blood-brain barriers. ABCG2 is responsible for the transport of several cancer therapeutic drugs. In cancer cells, as with other ABC transporters, ABCG2 is highly expressed, resulting in reduced drug concentrations inside the cell and consequent drug resistance^[94]^. Increased ABCG2 expression has been linked to resistance to MX, topotecan, 7-ethyl-10-hydroxycamptothecin, anthracycline and tamoxifen in breast cancer^[95-97]^. In a microarray analysis approach Jiao *et al*.^[98]^ showed that miR-181a is significantly downregulated while ABCG2 is overexpressed in MCF-7 cells resistant to MX. Using the same cells, the authors transfected them with miR-181a which abrogated ABCG2 expression and sensitized MCF-7 MX-resistant cells to MX. Furthermore, in an *in vivo* approach, an intra-tumoral injection of miR-181a mimics inhibited ABCG2 expression, and enhanced the antitumor activity of MX. Other authors have also shown that ABCG2 is regulated by miR-328^[99,100]^, miR-519c^[99]^ and miR-487a^[101]^ in breast cancer and influences MX resistance. Li *et al*.^[99]^ also showed that differences in expression of miR-519c and miR-328 are evident in stem cell-like ABCG2+ cells and their ABCG2- counterparts. Thus, new insights about drug resistance can be discovered by investigating miRNAs regulation in stem cells. In fact, more recently, several studies have been published showing the effect of some miRNAs in cisplatin resistance by modulating the ABCG2 transporter in stem cells, more precisely, miR-199a/b in colorectal cancer stem cells through the Wnt/β-catenin pathway (by directly targeting Gsk3β), and the authors showed that miR-199a/b is over-expressed in ALDHA1+ (primary colorectal cancer stem cells) and contribute to cisplatin resistance. Thus, here the authors showed that the effect on ABCG2 is not direct^[102]^. Other authors reported that miR-132 modulates cisplatin resistance by targeting the *SIRT1* gene in gastric cancer stem cells^[103]^. SIRT1 is a known upstream regulator of ABCG2 and the authors showed an inverse correlation between miR-132 and SIRT1 in gastric cancer tissues. Jia *et al*.^[104]^ showed that miR-3163 inhibits cisplatin resistance in retinoblastoma cancer stem cells. The authors validated ABCG2 as a target of miR-3163 and ABCG2 expression decreased significantly after ectopic overexpression of miR-3163. Finally, Guo *et al*.^[105]^ reported a miR-495/UBE2C/ABCG2/ERCC1 axis as modulator of cisplatin resistance in non-small cell lung cancer cells. Once again, miR-495 is not a direct regulator of ABCG2 but instead regulates UBE2C that has ABCG2 and ERCC1 as downstream targets.

## Conclusion

The intricate circuitry of miRNAs, the post-transcriptional regulation they mediate and their possible role in CDR is still far from being clarified in all its aspects and consequences. Epigenetic regulation, particularly by miRNAs, besides DNA methylation or histone acetylation, apparently has an important role not only in carcinogenesis but also in cancer treatment. The more than 2000 different human microRNA species identified thus far are part of an intertwined network of concurrently regulated proteins that mediate cell survival upon a challenge by cancer drugs by controlling the levels of expression of genes coding for metabolizing enzymes and transporter proteins as outlined above, being thus connected with drug resistance. Besides miRNAs, also long non-coding RNAs may regulate mRNAs’ levels of expression correlated with CDR. The fine tuning of the non-coding-RNAs system is also regulated by hypermethylation making the whole of the epigenetics a self-regulated system whose overall implications in CDR are yet to be fully uncovered but can hardly be considered as non-players in CDR and cancer therapy failure^[1]^.

## Declarations

### Copyright

© The Author(s) 2019.
